# Single-Molecule and Vesicle Trafficking Analysis of Ubiquitination Involved in the Activity of Ammonium Transporter AMT1;3 in *Arbidopsis* under High Ammonium Stress

**DOI:** 10.3390/cells11223651

**Published:** 2022-11-17

**Authors:** Ran Zhao, Yangyang Cao, Yanrui Ge, Jing Xu, Ruofan Li, Mei Yang, Yingying Chen, Dingjie Wu, Jianwei Xiao, Ruili Li

**Affiliations:** 1 National Engineering Research Center of Tree Breeding and Ecological Restoration, College of Biological Sciences and Biotechnology, Beijing Forestry University, Beijing 100083, China; 2 Institute of Tree Development and Genome Editing, Beijing Forestry University, Beijing 100083, China; 3 Shijiazhuang Zhonghua Avenue Primary School, Shijiazhuang 050000, China

**Keywords:** AMT1;3, ubiquitylation, dynamics, endocytosis, vesicle trafficking

## Abstract

Plants absorb nitrogen from the soil using ammonium transporters (AMTs). Plants can precisely regulate AMT1;3 levels using sophisticated regulatory systems, ensuring adequate nitrogen uptake without hazardous ammonium production. Here, we demonstrated that ubiquitylation can contribute to AMT1;3 degradation under high ammonium stress. Using the ubiquitin site mutant AMT1;3_K75R,K233R_-EGFP, we demonstrated that the loss of ubiquitination affects the dynamic characteristics of AMT1;3 proteins on the plasma membrane and markedly inhibits the endocytosis of AMT1;3 proteins under high ammonium stress. AMT1;3_K75R,K233R_-EGFP plants also showed inhibition of protein degradation that targets the vesicular pathway after being exposed to high levels of ammonium. Our findings showed that the dynamic properties, endocytosis, and vesicle trafficking pathways of AMT1;3 proteins are altered in AMT1;3_K75R,K233R_-EGFP under high ammonium conditions.

## 1. Introduction

Nitrogen (N), as one of the essential macronutrients required for plants, is a constituent of compounds such as proteins, nucleic acids, and cofactors [1,2]. For organic nitrogen, nitrate N and ammonium N are the predominant absorption forms in plants [1]. During plant growth and development, roots rely on nitrogen transporters to extract nitrogen from the soil and transport it to plant organs and cells [2]. In well-ventilated soil, nitrification of plants is efficient, and nitrate N is the main form of nitrogen for absorption [3]. During this process, plants depend largely on nitrate transporters (NRTs) to transport N [4]. However, under low temperatures and in flooded and acidic soils, ammonium N is the main nitrogen source absorbed by plants [5]. By the same token, ammonium transporters (AMTs) are important carriers for transporting ammonium nitrogen [6]. Because *Arabidopsis* is an ammonia-loving plant, when the concentrations of ammonium and nitrate are the same as in the external environment, *Arabidopsis* can absorb ammonium N at a rate 20 times that of nitrate N [7]. Therefore, to ensure the normal growth and development of plants, it is essential to understand the subtle regulatory mechanisms of ammonium uptake and transport.

The AMT family consists of six members in *Arabidopsis*, among which AMT1;3 is specifically expressed only in the roots [8,9]. Previous studies suggested that AMT1;3 exists in the form of an oligomer (monomer or dimer) at normal ammonium concentrations, but under high ammonium stress, AMT1;3 can exist in the form of a trimer and facilitate rapid endocytosis to prevent the accumulation of toxic levels of ammonium [10]. At the same time, a phosphorylation site mutation of AMT1;3 was found to affect the transport of ammonium ions, implying that phosphorylation modification is an important way of regulating the activity of ammonium transporters [11]. Therefore, the role of post-translational modification (PMT) of proteins in plant growth and development should not be neglected.

Ubiquitination functions as a molecular signal in a variety of biological activities, including protein degradation, membrane transport, and complex formation [12]. Ubiquitin is an 8.5-kDa protein that ubiquitin ligases use to bind to target proteins’ lysine (K) residues. Ubiquitin’s seven K residues and one N-terminal methionine residue allow it to form chains. This results in a wide range of topologically distinct ubiquitin chain types that influence a variety of cellular process [13]. Although the core concept of ubiquitin-dependent degradation of membrane proteins is conserved in plants, only a few membrane proteins have been shown to mediate endocytosis by ubiquitination. Mono-ubiquitination of the plant metal transporter IRON-REGULATED TRANSPORTER1 (IRT1), for example, is required for its constitutive turnover from the plasma membrane to the trans-Golgi network/early endosome (TGN/EE); ubiquitination extension into K63-linked chains in the presence of excess noniron metal ions leads to vacuolar degradation [14,15]. Furthermore, ubiquitination is involved in the endocytosis of the auxin efflux carrier PIN FORMED2 (PIN2) and the boron transporter REQUIRES HIGH BORON1 (BOR1) [16,17,18].

In the present study, we produced transgenic *Arabidopsis* plants with ubiquitination site mutations and performed the analysis of the distribution of AMT1;3 proteins on the plasma membrane. We examined the dynamics, endocytosis, and vesicle transport under high ammonium stress using live cell microscopy combined with biochemical and molecular biological techniques. Our results provide new insights into the ubiquitination involved in the regulation of ammonium transporter function. The findings will encourage further research that should aid in the establishment of nitrogen-efficient crop varieties from the biological level.

## 2. Materials and Methods

### 2.1. Plant Material and Construct

The *PRO_AMT1;3_:AMT*1;3*-EGFP* (GFP variant, monomeric enhanced green fluorescence protein) transgenic *Arabidopsis thaliana* in a Columbia-0 (Col-0) background was obtained from the laboratory of Lixing Yuan (China Agricultural University, Beijing, China). This is how the *AMT1;3_K75R,K233R_-EGFP* vector was made. We predicted that lysine (K) residues 233 and 75 may be ubiquitinated by the website (https://services.healthtech.dtu.dk/service.php?TMHMM-2.0) (Figure S1). After that, three gene fragments were amplified using the cDNA created through reverse transcription with primers (F1: 5′-GGGGTACCATGTCAGGAGCAATAAC-3′, R1: 5′-GCGGGCTCTAACAGAACCAG-3′; F2: 5′-AATACGATGAACATCATGC-3′, R2: 5′-GCGCTCGAACCGACCACG-3′, and F3: 5′-GGTGGTCGCGCTATTGCTCTG-3′, R3: 5′-TGCTCTAGATTAAACGCGAGGAGGAG-3′). The three gene fragments were then joined together by T4-DNA ligase forming a single AMT1;3 gene fragment, which was then subcloned as a *Kpn* I-*Xba* I fragment into an altered *pCAMBIA*1300-35*S-EGFP* vector.

WT plants were transformed with the *pCAMBIA*1300*-AMT*1;3*_K75R,K233R_-EGFP* expression vector by the *Agrobacterium tumefaciens*-mediated floral dip method [19], and transgenic seedlings were selected on half-strength MS solid medium (1% agar) containing 70 mg/mL hygromycin for mutants. Transgenic *Arabidopsis thaliana* seeds expressing AMT1;3-EGFP, AMT1;3_K75R,K233R_-EGFP, VHA-al-mRFP, AtSNX1-mRFP, and mRFP-SYP22 were described previously [20]. The AMT1;3-EGFP, AMT1;3_K75R,K233R_-EGFP, VHA-al-mRFP, AtSNX1-mRFP, and mRFP-SYP22 transgenic *Arabidopsis thaliana* were crossed with *PRO_AMT_*_1;3_*::AMT*1;3*-EGFP*, and AMT1;3_K75R,K233R_-EGFP seeds in the corresponding mutant background were obtained.

### 2.2. Growth Conditions

Transgenic *Arabidopsis thaliana* seeds in this study were surface-sterilized with an ethanol and H_2_O_2_ mixture (85% ethanol: 30% H_2_O_2_ = 4:1) and sown onto half-strength Murashige and Skoog (MS) medium containing 1% (*w*/*v*) sucrose and 0.8% (*w*/*v*) agar. After vernalization for two days at 4 °C, the seedlings grew at 25 °C with a 16 h light/8 h dark cycle for four days.

The high ammonium medium contained 1 mM KH_2_PO_4_, 1 mM MgSO_4_, 250 μM K_2_SO_4_, 250 μM CaCl_2_, 100 μM Na-FeEDTA, 50 μM KCl, 50 μM H_3_BO_3_, 5 μM MnSO_4_, 1 μM ZnSO_4_, 1 μM CuSO_4_, 1 μM NaMoO_4_ and 30 mM NH_4_NO_3_ (pH adjusted to 6.0 with KOH).

### 2.3. Western Blot Analysis

For Western blot analyses, total proteins were extracted from 14-old-day seedlings of transgenic AMT1;3-EGFP lines in the WT background under 60 min treatment of high ammonium (30 mM NH_4_NO_3_) with 50 μM CHX, after which 100 μM MG132 was added to one tube and DMSO medium was added to the other tube as a control. The protein concentration detection used monoclonal anti-GFP horseradish peroxidase-coupled antibodies. The AMT1;3-EGFP protein was denatured in Buffer Z (0.125 M Tris–HCl, pH 6.8; 2 mL glycerol; 2.4 g SDS; 4.4 mL β-mercaptoethanol; 0.2 mg bromophenol blue), separated on 10% (wt/vol) SDS/polyacrylamide gels, and transferred to a nitrocellulose membrane by electroblotting. Next, the membranes were washed with Tween-20 TBS (100 mL 10× TBS; 500 μL Tween20, and ddH_2_O added to a volume of 1 L) three times and incubated with the abovementioned antibodies. After several washes, the membranes were incubated in ECL chemiluminescence solution A with ECL chemiluminescence solution B in equal proportion. Finally, chemiluminescence was revealed using an Imaging System.

### 2.4. Confocal Laser Scanning Microscopy and Image Analysis

Four-day-old seedlings were transferred to liquid medium with or without inhibitors and incubated. Samples were viewed with an Olympus FV 1200 laser-scanning confocal microscope. EGFP, mCherry, YFP, mRFP, and FM4-64 were, respectively, excited at 488, 587, 512, 584, and 514 nm. The Olympus FV10-ASW 3.0 software package and ImageJ software v1.53t (NIH) were used for image analysis.

### 2.5. VA-TIRF Microscopy and Single-Particle Fluorescence Image

*Arabidopsis* plantlets were grown in high ammonium medium for four days. Using an inverted microscope (IX-71; Olympus) equipped with a total internal reflection fluorescence illuminator and a 100X oil-immersion objective (Olympus; numerical aperture = 1.45), the samples were captured using the VA-TIRFM approach. The position of the EGFP-AMT1;3 particles was determined by computing the weighted-centroid with sub-pixel accuracy after locating the local maxima with a mask of 33 pixels. The calculation excluded any spot that had a peak pixel that was less than three pixels away from another location. The motion range, velocity, and diffusion coefficients were calculated using the SPT method, as previously described by Cui et al. [21]. A time-lapse series of up to 100 photos per sequence, captured using a 100 ms exposure time shutter speed, detected the single EGFP-AMT1;3 particles.

### 2.6. Fluorescence-Correlation Spectroscopy Analysis

On a Leica TCS SP5 FCS microscope with a 488 nm argon laser, an internal coupled correlator, and an Avalanche photodiode, FCS was performed. FCS was carried out in the point-scanning mode after acquiring pictures of cells in the transmitted light mode. The local concentration of the fluorophore number was altered by the diffusion of AMT1;3-EGFP molecules into and out of the focus compartment, which resulted in spontaneous fluctuation of the fluorescence intensity [21]. Finally, the specific function was used to calculate the relevant fluorescence correlation spectrum curve.

### 2.7. Heterologous Expression of AMT1;3-EGFP in Yeast

The fragment of *AtAMT*1;3 cDNA carrying a C-terminal EGFP coding sequence was amplified by PCR from plamised *pCAMBIA*1300*-AMT*1;3*_K_*_75*R,K*233*R*_*-EGFP*, and then, the entry clone pDON-AMT1;3-EGFP was created by performing a BP recombination reaction (Invitrogen). We performed an LR recombination reaction to mobilize the fragment AMT1;3-EGFP into the pDR vector (according to the Gateway Technology manuals). The pDR-AMT1;3_K75R,K233R_-EGFP and the pDR-AMT1;3 (a gift from the laboratory of Wolf Frommer (Carnegie Institution for Science, Stanford, CA)) vectors were transformed into the triple MEP deletion yeast strain 31019b (mep1D mep2D mep3D ura3) by the lithium acetate method. Transformants were selected on minimal medium lacking uracil. Growth complementation assays were performed on solid yeast nitrogen base (YNB) medium (without an N source; BD Biosciences, BD233520) supplemented with 2% (wt/vol) galactose.

## 3. Results

### 3.1. Ubiquitination Is Correlated with the Degradation of AMT1;3 under High Ammonium Stress

**Figure 1:**
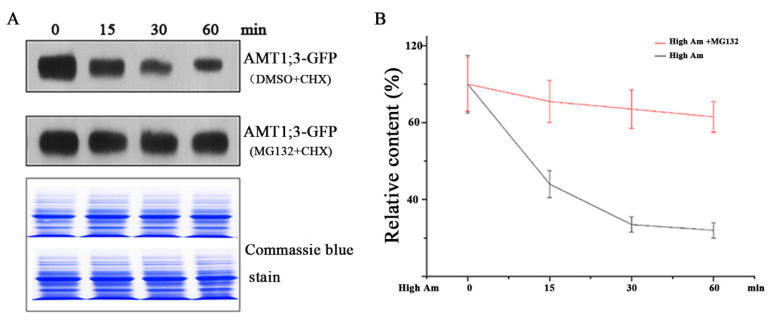
Ubiquitylation participates in the degradation of AMT1; 3 proteins under high ammonium stress. (**A**) Total proteins were isolated from AMT1;3-EGFP seedlings that had been treated with DMSO and MG132, in the presence of CHX, with high ammonium treatment for 0, 15, 30, 60 min. The total proteins were separated by SDS-PAGE and immune detected with GFP antibody. (**B**) Relative contents of AMT1;3-EGFP (per unit of total protein). Error bars represent standard errors.

Ubiquitination plays critical roles in regulating the activities of various proteins; it leads to proteins targeted to the 26S proteasome for degradation [12,22]. To examine the level of AMT1;3 protein, we pretreated the AMT1;3-EGFP seedlings for 60 min with the translation inhibitor cycloheximide (CHX), followed by high ammonium treatment for 0, 15, 30, and 60 min, and extracted total proteins from the transgenic seedlings. Immunoblot analysis revealed that the protein level of AMT1;3-EGFP decreased gradually after high ammonium treatment (Figure 1A), indicating that AMT1;3-EGFP was degraded in a time-dependent manner. However, treatment with MG132, a proteasome inhibitor, significantly inhibited AMT1;3 degradation (Figure 1A), indicating that the 26S proteasome is involved in AMT1;3 degradation. Figure 1B depicts the change in protein content. In short, these results suggest that ubiquitination under high ammonium stress is correlated with the degradation of AMT1;3 in *Arabidopsis*.

### 3.2. Ubiquitination Affects the Dynamics of AMT1;3 under High Ammonium Stress

To examine the effect of ubiquitination modification on AMT1;3 protein dynamics under high ammonium stress, we firstly predicted that ubiquitination would occur at lysine (K) residues 233 and 75 (Figure S1) and obtain the AMT1;3_K75R,K233R_-EGFP mutant. Confocal imaging showed that AMT1;3_K75R,K233R_-EGFP localized at the plasma membrane in root epidermal cells (Figure S2A). Furthermore, we verified the function of the AMT1;3_K75R,K233R_-EGFP by a complementation assay in yeast (Figure S2B), suggesting that AMT1;3_K75R,K233R_-EGFP maintained a normal ammonium transport capacity.

**Figure 2:**
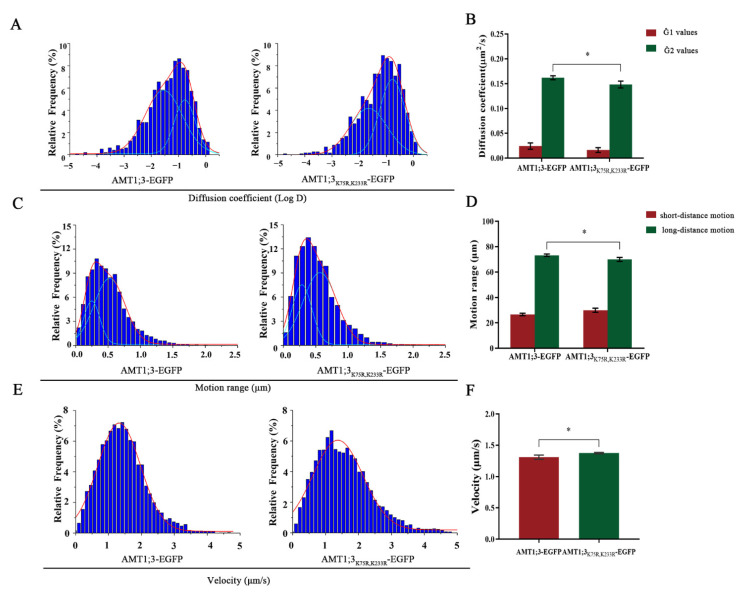
Ubiquitylation participates in the degradation of AMT1;3 proteins under high ammonium stress. (**A**,**B**) The distribution coefficient statistics of AMT1;3 and AMT1;3_K75R,K233R_-EGFP proteins. (**C**,**D**) Motion range of AMT1;3 and AMT1;3_K75R,K233R_-EGFP in *Arabidopsis*. (**E**,**F**) Distribution of AMT1;3 and AMT1;3_K75R,K233R_-EGFP velocity on the cell plasma membrane. * *p* < 0.05, *t* test. Error bars represent the mean ± SD.

Then, we used high ammonium liquid medium to treat AMT1;3-EGFP seedlings and AMT1;3_K75R,K233R_-EGFP seedlings for 30 min. We plotted the distribution of the diffusion coefficients on histograms and fitted the histograms using the Gaussian function, where we defined the Gaussian peaks (noted as Ĝ) as the characteristic values. The AMT1;3-EGFP seedlings’ Ĝ values were 2.43 ± 0.64 × 10^−2^ and 1.62 ± 0.04 × 10^−1^ μm^2^/s, while the AMT1;3_K75R,K233R_-EGFP seedlings’ Ĝ values were 1.63 ± 0.49 × 10^−2^ and 1.48 ± 0.07 × 10^−1^ μm^2^/s (Figure 2A,B), suggesting that the diffusion coefficient in the case of high ammonium was significantly decreased in AMT1;3_K75R,K233R_-EGFP seedlings. Therefore, we concluded that ubiquitination may change AMT1;3 diffusivity in response to high concentrations of ammonium.

We subsequently measured the range of motion of the AMT1;3 protein in AMT13-EGFP and AMT1;3_K75R,K233R_-EGFP seedlings. We defined the subgroup with small diffusion displacement as short-distance diffusion and the subgroup with large diffusion displacement as long-distance diffusion. In AMT1;3-EGFP seedlings, the motion range of AMT1;3 showed a bimodal distribution: long-distance motion (73.31%, 0.53 ± 0.02 μm, the Gaussian peak value) and short-distance motion (26.69%, 0.25 ± 0.01 μm, the Gaussian peak value) (Figure 2C). However, in AMT1;3_K75R,K233R_-EGFP seedlings, long-distance motion decreased to 69.98%, and short-distance motion increased to 30.02% (Figure 2D). This indicated that ubiquitination may extend the AMT1;3 protein diffusion range to respond to stress.

Finally, we studied the movement velocity of AMT1;3 in the root elongation region under ammonium stress. In AMT1;3-EGFP seedlings, the value of Ĝ was 1.31 ± 0.03 μm/s. In AMT1;3_K75R,K233R_-EGFP seedlings, the Ĝ value was 1.38 ± 0.01 μm/s (Figure 2E,F). The movement velocity was increased in AMT1;3_K75R,K233R_-EGFP, indicating that mutation of the ubiquitination site significantly accelerated the movement of the AMT1;3 protein under high ammonium stress.

### 3.3. Loss of AMT1;3 Ubiquitination Impairs Endocytosis under High Ammonium Stress

**Figure 3:**
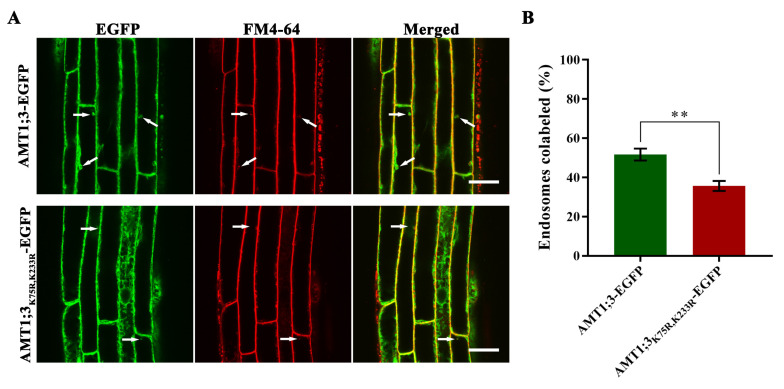
The ubiquitinated forms of AMT1;3 proteins affect its endocytosis. (**A**) The partial colocalization of AMT1;3/AMT1;3_K75R,K233R_-EGFP with FM4-64. The left photographs represent GFP signal showing subcellular localization of AMT1;3/AMT1;3_K75R,K233R_-EGFP. The middle photographs represent red fluorescence signal showing the plasma membrane stained by FM4-64. The right photographs represent the merge of AMT1;3/AMT1;3_K75R,K233R_-EGFP and FM4-64 staining. Scale bars = 20 µm. White arrows indicate the colocalization spots of AMT1;3/AMT1;3_K75R,K233R_-EGFP and FM4-64. (**B**) Statistics of the colocalization of AMT1;3/AMT1;3_K75R,K233R_-EGFP with FM4-64. ** *p* < 0.01, *t* test. Error bars represent the mean ± SD.

To clarify whether ubiquitination affects endocytosis of the AMT1;3 protein under high ammonium stress, we firstly used high ammonium liquid medium to treat AMT1;3_K75R,K233R_-EGFP seedlings and AMT1;3-EGFP seedlings for 30 min, then added FM4-64 and treated for another 30 min. The co-localization ratios of AMT1;3-EGFP protein and FM4-64 in AMT1;3-EGFP plant are 51.67%, while AMT1;3_K75R,K233R_-EGFP protein and FM4-64 in AMT1;3_K75R,K233R_-EGFP plant are 35.67% (Figure 3A,B). The results showed that the loss of AMT1;3 ubiquitination impaired endocytosis under high ammonium stress.

We further probed the density of ammonium transporters on the plasma membrane under ammonium stress. We found that the molecular density was 16.73 ± 3.97 and 30.74 ± 4.70 N/µm^2^ in AMT1;3-EGFP seedlings and AMT1;3_K75R,K233R_-EGFP seedlings, respectively (Figure S3). These results suggested that ubiquitination may trigger endocytosis of AMT1;3 under high ammonium concentration, thereby avoiding excessive absorption of NH_4_^+^ ions.

### 3.4. Ubiquitination Affects Vesicular Transport of the AMT1;3 Protein under High Ammonium Stress

Previous studies have shown that when exogenous substances enter the cell, they first enter the early endosome, then the late endosome, and finally the vacuole for degradation. To study the vesicle transport pathway of the ubiquitination site-mutated AMT1;3 protein, we used the emasculated hybridization method to obtain hybrid transgenic plants labeled with a two-color fluorescent protein, i.e., AMT1;3-EGFP and AMT1;3_K75R,K233R_-EGFP plants were hybridized with VHA-a1-mRFP, AtSNX1-mRFP, and mRFP-SYP22. We used BFA and Wortmannin (an inhibitor of phosphatidylinositol-3 kinase) to treat the three groups of plants mentioned above for 60 min. These results showed that even after the ubiquitination site is mutated, the proteins still transported to the early endosome, late endosome, and the vacuole (Figure S4).

**Figure 4:**
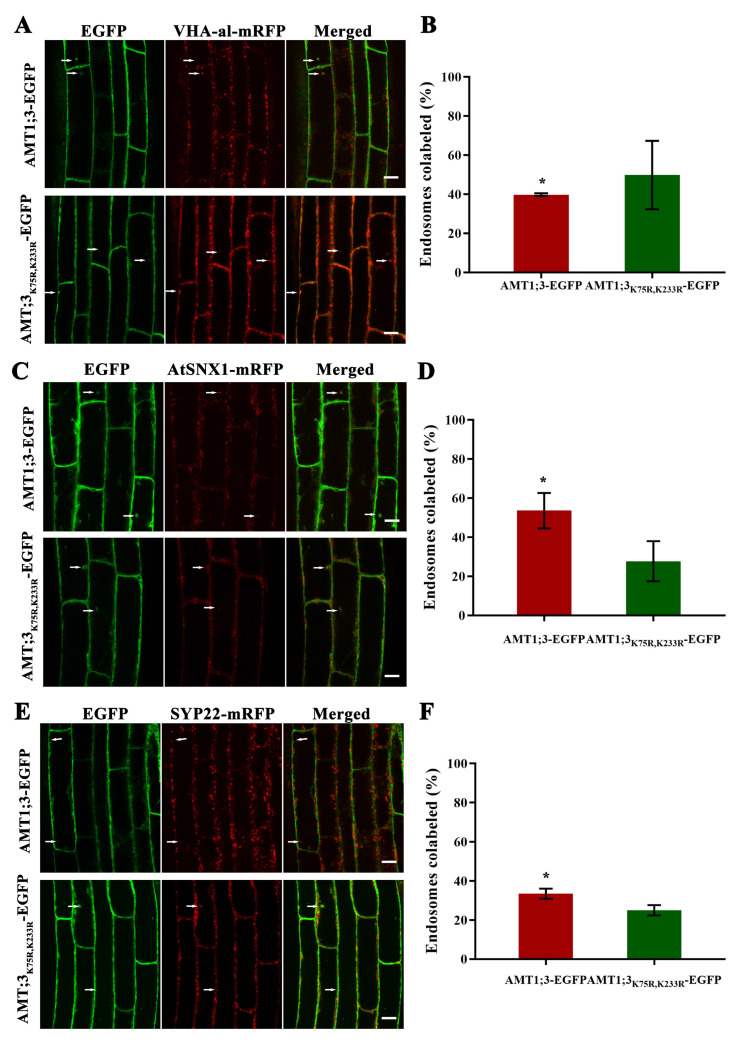
Ubiquitylation affects the colocalization of AMT1;3 with vesicle-transport-labeled proteins under high ammonium stress. (**A**) VHA-a1-mRFP endosomal markers partially colocalized with AMT1;3/AMT1;3_K75R,K233R_-EGFP. Colocalizing endosomes are indicated by white arrows. AMT1;3/AMT1;3_K75R,K233R_-EGFP is shown in green, VHA-a1-mRFP in red, and the merged image indicates colocalization (yellow). (**B**) Statistics of the colocalization of AMT1;3/AMT1;3_K75R,K233R_-EGFP with VHA-a1 proteins. (**C**) AMT1;3/AMT1;3_K75R,K233R_-EGFP partially colocalizes with late endosomal markers. The AMT1;3/AMT1;3_K75R,K233R_-EGFP is shown in green, the AtSNX 1-mRFP is shown in red, and the merged image shows colocalization (yellow). (**D**) Statistics of the colocalization of AMT1;3/AMT1;3_K75R,K233R_-EGFP with AtSNX1 proteins. (**E**) SYP22-mRFP vacuole markers partially colocalized with AMT1;3/AMT1;3_K75R,K233R_-EGFP. Colocalizing endosomes are indicated by white arrows. AMT1;3/AMT1;3_K75R,K233R_-EGFP is shown in green, SYP22-mRFP in red, and the merged image indicates colocalization (yellow). (**F**) Statistics of the colocalization of AMT1;3/AMT1;3_K75R,K233R_-EGFP with SYP22 proteins. Scale bars = 10 μm. White arrows indicate endosomes showing colocalization. * *p* < 0.05, *t* test. Error bars represent the mean ± SD.

We further studied the effect of ubiquitination on the vesicle transport pathway of AMT1;3. Confocal imaging showed that AMT1;3-EGFP/AMT1;3_K75R,K233R_-EGFP partially colocalize with VHA-a1-mRFP; further statistics showed that the proportion of colocalization of the AMT1;3-EGFP protein and VHA-al protein is 39.8%. After the mutation of the ubiquitination site, the proportion of co-localization increased to 49.9% (Figure 4A,B). Compared to the AMT1;3-EGFP protein, the proportion of AMT1;3_K75R,233R_ protein with AtSNX1 protein colocalization decreased significantly (from 53.7% to 27.8%) (Figure 4C,D). In a similar way, loss of ubiquitination results in the proportion of colocalization of AMT1;3_K75R,K233R_-EGFP with mRFP-SYP22 being reduced significantly (from 33.5% to 25.1%) (Figure 4E,F). These findings revealed that ubiquitination loss accelerates the accumulation of the AMT1;3 protein in the early endosome and inhibits AMT1;3 accumulation in the late endosome as well as AMT1;3 degradation in the vacuole.

## 4. Discussion

The ammonium transporter (AMT) is the most important protein responsible for the uptake of ammonium nitrogen in plants. With the development of plant genomics, increasing numbers of AMT genes have been detected. There are at least 10 AMT members in the rice genome, including *OsAMT*1 to *OsAMT*4 [23]. Different amounts of AMT proteins have also been identified in *Saccharum* and *Malus domestica* [24,25]. In *Arabidopsis*, AMT1;3 is a member of the AMT family that is only expressed in the roots. Previous studies have shown that the clustering and endocytosis of AMT1;3 provides an effective mechanism by which plant cells can avoid the accumulation of toxic levels of ammonium, and its T464, phosphorylation at the C-terminal region (CTR^C^) acts as a prime switch to prevent excess ammonium influx [26,27]. However, not much is known about the ubiquitination that regulates the mechanism of endocytosis and vesicle trafficking of AMT1;3 and affects the dynamic characteristics of the protein under high ammonium stress. In this report, we examined the distribution of AMT1;3 proteins on the plasma membrane, its movement dynamics, and its roles in endocytosis and vesicle transport under high ammonium stress.

Ubiquitination is a reversible posttranslational modification of cellular proteins. This process has been shown to be required for the entry of certain cargo proteins into vesicles at different stages of the secretory/endocytic pathway [28]. Previous research has shown that PIP2;1, PIN2, and FLS2 are stabilized following MG132 treatment [29,30,31]. In our study, we showed that the degree of protein degradation was significantly inhibited when MG132 was added, indicating that ubiquitination may regulate degradation of the AMT1;3 protein

Diverse forms of ubiquitination are involved in the distinct membrane protein degradation pathways in yeast and mammalian systems [32,33,34,35]. For example, using various biochemical and molecular genetic approaches, researchers discovered that a single ubiq-uitin is sufficient for receptor tyrosine kinases (RTK) internalization and degradation [33]. In plants, ubiquitin is also able to lead artificial PM proteins into the endocytic pathway and facilitate sorting into the vacuole for degradation [36]. Recent study showed that mono-ubiquitination of the membrane-associated receptor-like cytoplasmic kinase BOTRYTIS-INDUCED KINASE1 (BIK1) triggers its endocytosis [37]. Our data also show that the partial co-localization ratio of the AMT1;3 protein decreased significantly, while its molecular density increased significantly in the AMT1;3_K75R,K233R_-EGFP plant, implying that ubiquitination may trigger endocytosis of AMT1;3 under high ammonium conditions. Studies in yeast and mammals have demonstrated that ubiquitination occurs at various steps in endocytosis to control plasma membrane protein internalization and sorting into internal vesicles of MVBs/LEs on their way to the vacuole/lysosome [38]. By observing the co-localization of the AMT1;3 protein with three organelle-labeled proteins, we found that the loss of the ubiquitination site led to the accumulation of the AMT1;3 protein in the early endosomes and inhibition of transport to the late endosomes, ultimately leading to a reduction of protein degradation in vacuoles under high ammonium stress. Similar results have been reported for BRI [39].

Further studies are needed on the enzymes E1, E2, and E3 associated with the ubiquitination of AMT1;3 and the specific degradation process. Whether the ubiquitination of the AMT1;3 protein in *Arabidopsis* is mono-ubiquitination or polyubiquitination remains to be further explored. In addition, the main endocytosis pathway of the AMT1 protein in the absence of ubiquitination sites needs to be further explored using a combination of biological technologies.

## 5. Conclusions

The data presented here showed that ubiquitination is involved in degradation of the AMT1;3 protein under high ammonium stress. More specifically, ubiquitination may change AMT1;3 diffusivity, diffusion range, and movement. Furthermore, ubiquitination may trigger endocytosis and alter vesicular transport of the AMT1;3 protein. Whether there are other protein degradation pathways is a question that will be addressed in future studies.

## Data Availability

Not applicable.

## Supplementary Materials

The following supporting information can be downloaded at: https://www.mdpi.com/article/10.3390/cells11223651/s1, Figure S1: Topological structure of the AMT1;3 ammonium transporter; Figure S2: Identification of transgenic plants; Figure S3: The density of AMT1;3-EGFP and AMT1;3_K75R,K233R_-EGFP molecules as acquired with FCS.; Figure S4: AMT1;3/AMT1;3_K75R,K233R_-EGFP and vesicle-transport-labeled proteins partially colocalized in elongation zone of transgenic *Arabidopsis* roots.
