# Supplementary material for: Single-Molecule and Vesicle Trafficking Analysis of Ubiquitination Involved in the Activity of Ammonium Transporter AMT1;3 in Arbidopsis under High Ammonium Stress
Source: Cells. 2022 Nov 17;11(22):3651. doi: 10.3390/cells11223651 (PMC9688738; doi:10.3390/cells11223651)
Supplement: Supplementary file 1 [file cells-11-03651-s001.zip › cells-2014105-supplementary.pdf]

SUPPLEMENTARY MATERIALS

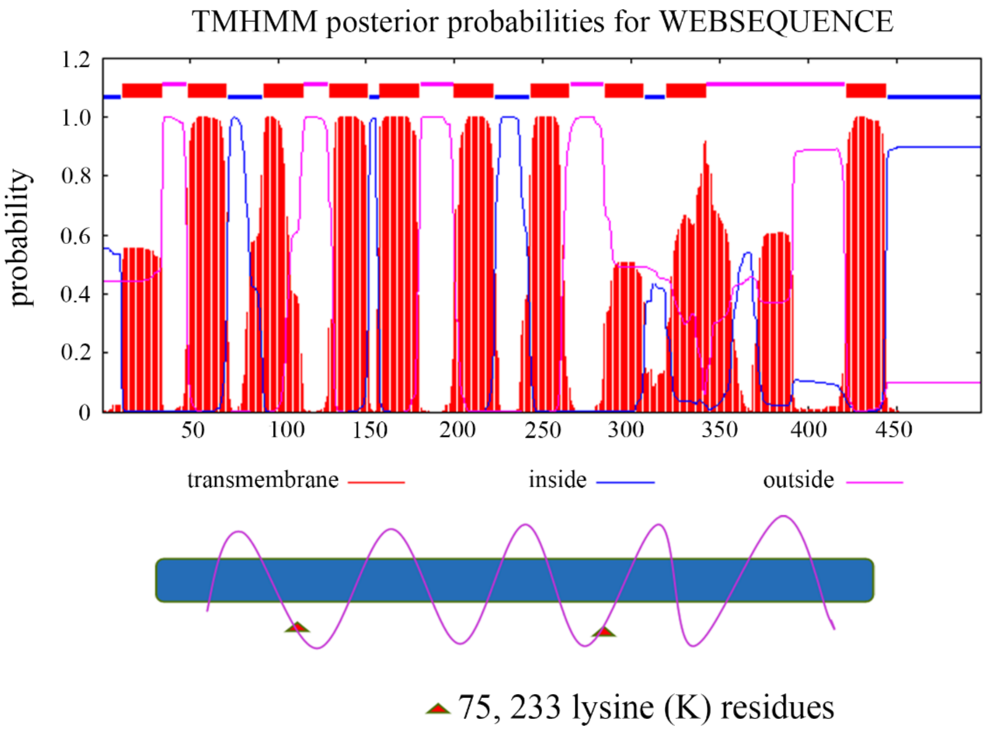

Figure S1 Topological structure of the AMT1;3 ammonium transporter. According to the topological structure, we predicted that lysine (K) at positions 75 and 233 would be ubiquitinated.

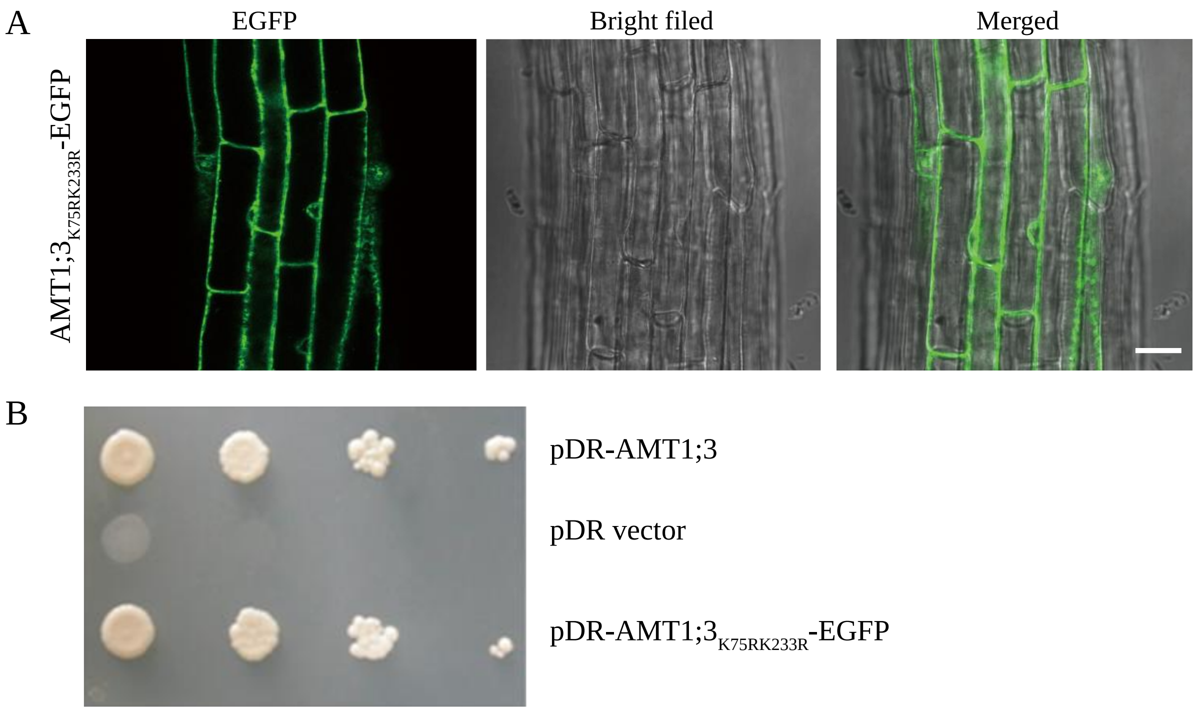

Figure S2 Identification of AMT1;3$_{K75R,K233R}$-EGFP plants. (A) Confocal imaging of AMT1;3$_{K75R,K233R}$-EGFP fusion proteins expressed in *Arabidopsis* roots. Scale bar = 20 μm. (B) Functional analysis of AMT1;3$_{K75R,K233R}$-EGFP in yeast. The triple MEP yeast mutant was transformed with the vectors pDR, pDR-AMT1;3, and pDR-AMT1;3$_{K75R,K233R}$ -EGFP. The results showed that both intact AMT1;3, and AMT1;3$_{K75R,K233R}$-EGFP restored growth of a yeast strain defective in ammonium uptake on medium. Cells transformed with an empty vector did not show restored growth. This suggests that AMT1;3$_{K75R,K233R}$-EGFP maintained a normal ammonium transport capacity.

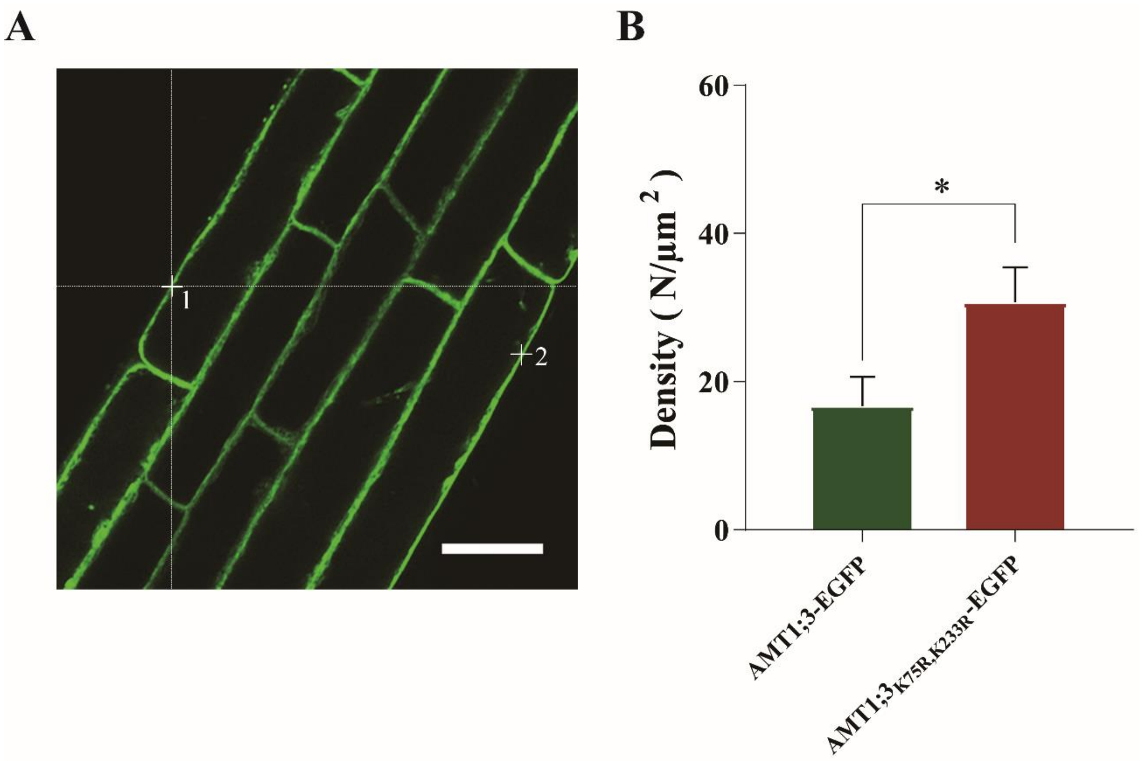

Figure S3 The density of AMT1;3-EGFP and AMT1;3$_{K75R,K233R}$-EGFP molecules as acquired with FCS. (A) The laser beam was focused on points 1 and 2 to monitor the fluorescence fluctuations. Scale bar = 20 μm. (B) The density of AMT1;3-EGFP and AMT1;3$_{K75R,K233R}$-EGFP particles on the plasma membrane of elongation zone cells. *p < 0.05, t-test. Error bars represent the mean ± SD.

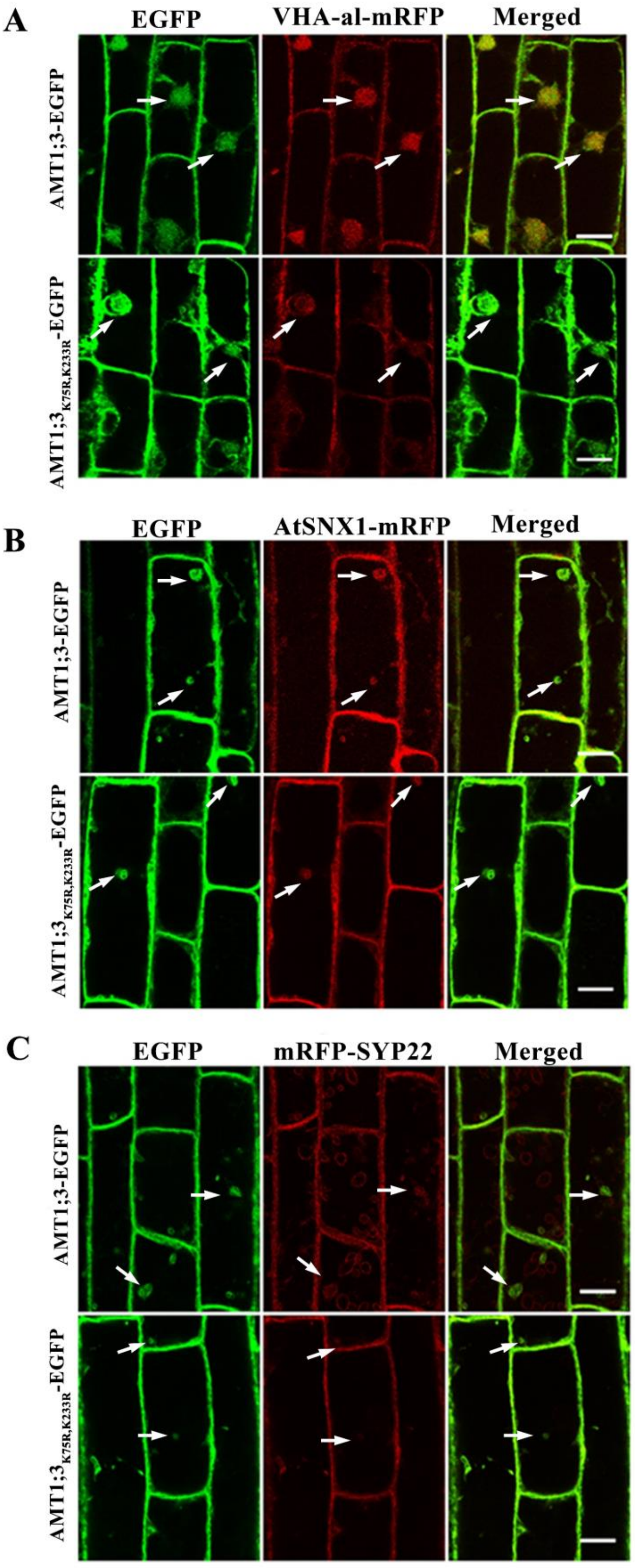
A
EGFP
VHA-al-mRFP
Merged
AMT1;3-EGFP
AMT1;3$_{K75R,K233R}$-EGFP
B
EGFP
AtSNX1-mRFP
Merged
AMT1;3-EGFP
AMT1;3$_{K75R,K233R}$-EGFP
C
EGFP
mRFP-SYP22
Merged
AMT1;3-EGFP
AMT1;3$_{K75R,K233R}$-EGFP

Figure S4 AMT1;3/AMT1;$3_{K75R,K233R}$-EGFP and vesicles transport labeled proteins partially colocalized in elongation zone of transgenic *Arabidopsis* roots. (A) AMT1;3/AMT1;$3_{K75R,K233R}$-EGFP partially colocalized with VHA-a1-mRFP endosomal markers. White arrows indicate endosomes showing colocalization. Before microscopy imaging, cells were treated with BFA (50 μM) for 1 h. (B) AMT1;3/AMT1;$3_{K75R,K233R}$-EGFP partially colocalizes with late endosomal markers. When cells were treated by incubation in Wortmannin (33 μM) for 1 h, AMT1;3/AMT1;$3_{K75R,K233R}$-EGFP partially colocalized with AtSNX1-mRFP (white arrows). (C) Partial colocalization of mRFP-SYP22 and AMT1;3/AMT1;$3_{K75R,K233R}$-EGFP (white arrows). For confocal microscopy analyses, inhibitors (33 μM Wortmannin) were applied in liquid 1/2 MS medium before imaging. Scale bars = 10 μm.
